# A Hybrid Methodology for the Evaluation of Clinical Practice in Final-Year Nursing Students

**DOI:** 10.3390/nursrep13030088

**Published:** 2023-07-25

**Authors:** Elsa Gil-Mateu, Silvia Reverté-Villarroya, Núria Albacar-Riobóo, Josep Barceló-Prats

**Affiliations:** 1 Nursing Department, Campus Terres de l’Ebre, Universitat Rovira Virgili, Avenue Remolins, 13–15, 43500 Tarragona, Spain; elsa.gil@urv.cat (E.G.-M.); nuria.albacar@urv.cat (N.A.-R.); 2 Advanced Nursing Research Group, Universitat Rovira i Virgili, 43002 Tarragona, Spain; josep.barcelo@urv.cat; 3 Nursing Department, Campus Catalunya, Universitat Rovira Virgili, Avenue Catalunya, 35, 43002 Tarragona, Spain

**Keywords:** teaching methods, nursing students, nursing faculty practice, self-evaluation program

## Abstract

(1) Background: Clinical practice constitutes a scenario where the student approaches reality. The pedagogical relationship that is built between the nurse, the tutors and the student becomes important. And this requires intentional and reflective accompaniment. The principal objective was to design a hybrid-learner-centered training model requiring reflection and acquisition of specific skills. (2) Methodology: This was a prospective observational study using an intentional sample of 87 students. A hybrid model based on a dynamic virtual forum and Individual Improvement Plan (IIP) was constructed, evaluated using a self-completed questionnaire with a Likert scale. (3) Results: A model of accompaniment to the practices was built that allows for unifying a work plan. A transversal activity IIP was designed. A discussion forum was incorporated for each subject tutor. The analysis of the questionnaire showed that learning assessment, tutorials, virtual forums, self-assessment and satisfaction statistically differed. (4) Conclusions: The model allows students to be accompanied to acquire skills, knowledge, and attitudes and to develop critical thinking, as well as to improve the teaching quality of the practices of the Curriculum of the Nursing degree and to achieve their own competences through student-centered methodologies. This study was not registered.

## 1. Introduction

Clinical practice occupies an important place in the nursing degree curriculum, and students in Spain must perform a total of 2300 h of practice during their four academic years, with most of this concentrated in the final year [1]. Practical subjects are where most theoretical and methodological knowledge transfer takes place, and where all scientific, relational, ethical, attitudinal and procedural learning must actually be deployed. These skills are based on the development of six axes, which constitute the basis on which the learning process of clinical practice is based: professional values, attitudes, behaviors and ethics of care; the scientific foundations of the biological, human and social sciences; critical, logical and creative thinking; information and knowledge management; clinical communication and relationship skills; and, finally, procedural clinical skills [2]. Clinical practice is meant to be an open, dynamic and changing space characterized by the complexity of clinical situations, and some studies [3,4] indicate that students may experience high levels of stress, depression and anxiety, especially as they approach the final stages of their degree. Pulido-Martos et al. [5] show that among stress-inducing factors are the perceived lack of knowledge and skills needed to care for patients or having to work under the close supervision of tutors. Given this reality, undergraduate training must offer strategies and accompanying resources that help students to carry out their clinical practice requirements in an enabling environment. In this context, the pedagogical relationship that is built between the nurse, the tutors and the student becomes important. Intentional and reflective mentoring is required to guide the student’s own reflection based on their experience and training and to help them develop analytical and critical thinking [6]. Based on the affirmation from Alfaro-Lefevre [7], it is not a method to be learnt, but a changing process, that needs skills, knowledge and some attitudes or dispositions; it is contextual, has a target and looks for self-improvement.

In this sense, the tutor must seek resources for dialogue around the significance of the act of caring, linking the results of students’ reflective processes to future practice and encouraging reflective, scientific, dialogue-based and ethically competent actions. This learning, according to various authors [8], is generated when the student actively participates in its process, constructs meanings and elaborates proposals based on collaboration with both teachers and peers. Such learning thus occurs in situations that drive the student to develop their knowledge in a meaningful way. The aim for tutors is to make sense of each action and to link the results of this reflective process to future practice, i.e., to move from what to teach to how to teach, leading students to meaningful and deliberate learning. Schön [9] calls this process reflection in action. Although these skills are not necessarily innate, they can be learned through specific training, which is why nursing educational programs should contribute to the development of critical thinking skills. This is so much so that Zuriguel [10] proposes that educational institutions should structure syllabi, curricula, teaching methodologies and evaluation systems with the aim of integrating strategies that promote an educational environment that strengthens and develops these skill [11].

The type of learner, content to be studied, location of the participants in the process and electronic media available are all factors that will determine the model to be implemented. The literature consulted [11,12,13,14] shows that mixed learning models—known as “b-learning”—combine the best of virtual methods with face-to-face methods. However, in all of them, it is important to encourage the creation of a learning community—a space for the construction of knowledge where students and tutors can interact [15,16,17]. As a result, active methodologies such as forums, the flipped-classroom or conceptual maps are a strategic framework for student-centered learning, steering trainees toward action [18,19,20].

In the case of clinical practicums, it is difficult to find assessment tools that allow for an active methodology outside the classroom and that fit the philosophy of higher education. This study proposes a model based on these methodologies to accompany the student in their practicum and to be able to evaluate their competences. It is true that hybrid learning modalities are used in many countries, but in clinical practices outside the university, it is difficult to monitor the student.

Therefore, this study proposes a hybrid model based on two axes: the theory–practice relationship and critical thinking. The model follows a constructivist theoretical approach, understanding knowledge as a constructive-creative process where students are expected to play an active role, promoting an enquiring attitude to research and developing what is meaningful to them. Thus, the main objective of this study was to design a hybrid-learner-centered training model requiring reflection and acquisition of specific skills, which could be integrated into the clinical practice requirements of the nursing degree. A secondary objective was to evaluate the satisfaction with the proposed model among students in the last year of their nursing degree.

## 2. Materials and Methods

A prospective observational study was carried out at the Faculty of Nursing in the University Rovira i Virgili of Tarragona (URV), Terres de l’Ebre Campus (Tortosa, Spain).

### 2.1. Sample

A total and intentional population of 87 fourth-year nursing degree students were selected. They were enrolled in three clinical practice subjects of the second block of the academic year, which corresponds to the Curriculum (2009): Geriatric Care, Critical Care and Psychiatry and Mental Health during the 2018–2019 and 2019–2020 academic years. The subjects are divided into internship cycles of 5 weeks each, where in small groups, the students undergo 4 cycles of practices.

### 2.2. Ethical Considerations

The study obtained approval from the Pere Virgili Institute for Health Research’s Drug Research Ethics Committee, with the code 121/2020. All participants were duly informed and signed the corresponding informed consent voluntarily.

### 2.3. Instruments

**Figure 1:**
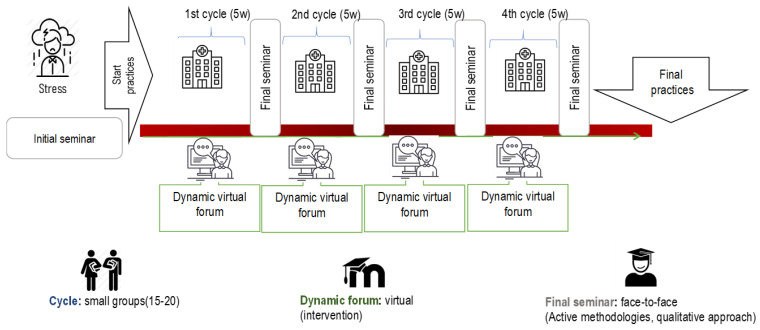
Hybrid model of learning in clinical practice.

The tools that constituted the hybrid model of learning in clinical practice were (Figure 1).

A self-completed questionnaire (ad hoc): This questionnaire was created by the authors and was based on the specific needs of collecting short-term satisfaction with the hybrid model of learning. The questions of the ad hoc questionnaire were based on the models of three authors, Cabero Almenara [21], de Milenca Vilca Pozo et al. [22] and Pineda Herrero et al. [23]. Information was collected using the ad hoc questionnaire, which included a total of 50 questions on a Likert-type scale scored from 1 to 4 (1 = strongly disagree, 2 = disagree, 3 = agree, and 4 = strongly agree). The instrument included sociodemographic variables and satisfaction with the hybrid model. The different sections of the ad hoc questionnaire did not form a construct among themselves. Three aspects were asked, such as the functioning of the forum, assessment of the tutor and satisfaction. The last three questions consisted of a self-assessment. The instrument was supervised by a group of experts composed of professors from the psycho-pedagogical areas of the same university. The relevance, adequacy and clarity of the questions were assessed to obtain the opinion of the students in relation to the different parts of the model.

Dynamic virtual forum: This was structured as the core of the model. The guidelines for the use, objectives and description of the activities were drawn up in advance. Training was given to the tutors who are facilitators of the forum. These are the academic professors coordinating the subjects and have been the key to developing the tool, as they served as reference points for the learner, be it to motivate, help and facilitate information or to redirect issues that arose and to keep the virtual environment alive.

The Individual Improvement Plan (IMP): this document was designed and structured (Table 1) based on a documentary analysis [24,25,26]. Evidence was collected to reflect the progress and objectives achieved by the students during their clinical placements [27,28]. The document allows for a grouping of improvement activities and interventions, coordinated and aggregated to avoid dispersion in their reflection [29]. In the introductory seminar of the practices, the document informed students about its structure, how to fill it in and its objective. At the end of the internship period, they will have to deliver it through Moodle: a virtual learning platform.

### 2.4. Design and Implementation

Three phases were established for implementing the hybrid model of student support during clinical placements:

- Phase 1 “Diagnostics”: A consolidated work plan with cross-curricular activities was designed. The Individual Improvement Plan (IMP) and support material were provided on the Moodle platform.
- Phase 2 “Training”: In the introductory seminar to the clinical placements and in the introductory seminar of the practices, the timetable of the model, that is, the dates of completion and delivery of the activities, was explained to the students. They also informed students of the IMP document, explaining its structure, how to complete it and the desired objectives. An asynchronous discussion forum was set up that would be run by the tutors of each subject for each internship period. At the end of each cycle, a face-to-face seminar was given where each student assigned a category code to their contributions to the forum and, in groups of 4 students, presented the results to the rest of the group by means of a concept map. Finally, they completed the model satisfaction questionnaire.
- Phase 3 “Summary”: Evaluation of the students’ learning during the forum contributions was continuous and individual. This activity was linked to the IMP, where students incorporated all those aspects discussed in the forum and during their clinical practice experience, being evaluated at the end of each period by the tutor in charge.

SPSS version 27.0 was used to analyze the questionnaire results (Statistical software, IBM Corp., Armonk, NY, USA). The level of significance was set at *p* < 0.05 and *p* < 0.001. Descriptive statistics were carried out on the sociodemographic characteristics of the total subjects included, and 3 groups of clinical practice, with means and standard deviations. The distribution of the data was evaluated using the Shapiro–Wilk normality test. An ANOVA was used to compare the 3 groups of clinical practice.

## 3. Results

### 3.1. Assessment of the Model

The implementation of the model resulted in a total of 261 IMPs: 87 for each subject. Each student delivered three IMPs, one for each subject. The sections of the Individual Improvement Plan document were based on reflection, action, evaluation, participation, personnel, usefulness and transfer [29].

In terms of participation across the six forums, there were a total of 1734 participations, with an average of 289 contributions per forum. In the Critical Care Unit placement, there were a total of 450 interventions with an average of 89 per forum. In the Geriatric Unit, there were 591 participations, with an average of 98.5. In the Mental Health Units, there were 693 participations with an average of 100 per forum. Finally, 30 concept maps were obtained, 13 for each subject.

### 3.2. Satisfaction Questionnaire: Analysis

A total of n = 158 (90.8%) self-completed questionnaires were obtained. Of the total, n = 111.5 (70.6%) of the students were women with a mean age of 21.18 years (SD = 2.186).

The analysis of the questionnaire showed mean scores above 3 points in almost all sections of the questionnaire, indicating a high degree of satisfaction in each of the questions. Likewise, when comparing the mean scores between the three clinical practice subjects, Geriatric Care, Critical Care and Psychiatry and Mental Health, significant differences were found. The first section collected the opinions in relation to the virtual learning environment and discussion forum: item 3 (*p* = 0.004), item 4, (*p* = 0.001), item 7 (*p* = 0.007), item 10 (0.008), item 11 (*p* < 0.001), and item 15 (*p* = 0.001). In the motivational tutor section of the forum: items 18 (*p* = 0.041), 23 (*p* < 0.001), 25 (*p* = 0.043), 28 (*p* = 0.001), and 29 (*p* = 0.016). Regarding overall satisfaction with the hybrid model, statistically significant differences were observed between the scores of item 45 (*p* < 0.001). Finally, no differences were observed in the scores of the self-assessment section (Appendix A, Table A1).

## 4. Discussion

The general results of the ad hoc questionnaire, in line with results from other studies [30], show that the forum and the virtual space were satisfactorily evaluated by the students.

We observed that the creation of an easily accessible virtual space, with no time restrictions and in small groups where experiences can be shared and explained, promotes debate and improves support by personalizing the supervision of students during their clinical training. This space is, therefore, ideal for recognizing and learning from previous experiences. Chan’s study [31], which reviews strategies to improve critical thinking, concludes that the forum is a space that allows interaction. The tutor, who is constantly with the students during this learning process, is in charge of reassuring them, grounding their reflections and helping them grow. However, we also observed some points of disagreement between the groups. Regarding tutors, elements to be improved were helping with collaborative learning, contributing with more reference sources, giving individual feedback and synthesizing information. The literature [32] has already shown that one of the greatest challenges for teachers who propose a forum is to encourage participation and keep students motivated, with importance given to creating environments where students feel stimulated. Different studies [33,34] have shown that feedback is of central importance in online training processes, especially in the types of learning that require some degree of personalization.

The differences observed in this study suggest that student motivation is crucial for the tool to be used correctly and to achieve the objectives of the model. It is evident—and existing studies corroborate this [35]—that social motivations such as the desire to share knowledge, personal motivations such as recognition from colleagues and teachers, and technological motivations such as the usability and convenience of the platform play a key role in the success of discussion forums.

Motivation arises when what is being discussed gains relevance and when friendly and challenging spaces are created, which generate conceptual discussions that encourage participants to seek knowledge [36]. Therefore, it is important to highlight the role of the tutor as a person who galvanizes the platform while creating a safe space. The fact that this model is organized in small groups helps with students’ motivation to share their experiences. It is a space to contribute to one’s experiences with profound reflection, allowing a university education to shape competent, critical, creative and reflective students. The role of the teacher is to facilitate, guide and mentor students during their training, promoting their autonomy and ability to reflect on and regulate their own learning [37].

Thus, there is no doubt as to the importance of well-developed tutorial action and the high level of responsibility that falls on the tutor during clinical practice placements, where students have a real opportunity to learn in a professional context, to assess their level of mastery in real situations and to have the potential to both reflect and act on what has been learned in the university.

The practicum allows students to gauge their competencies and not only knowledge or abilities. But the competency approach in clinical practice, as Morales and Varela [38] warn, still requires collaboration, exploration and a clear construction of its premises to be able to develop and implement precise strategies for its teaching and evaluation. Armengol et al. [39] argue that this moment should be used as an opportunity for students to make individualized work plans, allowing them to acquire the skills they do not yet have and to strengthen the ones they already possess.

Along these lines, various initiatives have been developed to introduce active methodologies. However, Canalejas et al. [40] show that the methods used have often enhanced reproductive learning, rather than knowledge construction. Our proposed model is presented as a space for students to reflect on their lived experiences during clinical practice, allowing for a university education that trains competent, critical, creative and reflective students. Reflection here is understood as a means to improve professional action, not as an end in itself.

Combining active methodologies creates a model of semi-virtual assistance, a resource for the exchange of knowledge among participants that, in the form of an educational strategy, makes it possible to encourage critical thinking in the world of clinical practice. Other studies [41,42,43] also show that active methodologies are instruments that not only improve critical thinking, but also increase academic performance and motivation.

All together, the model is able to enhance students’ reflections both during and on their clinical practice, acting as a guide that promotes critical thinking in a clinical practice environment where students often feel alone and do not perceive the relevance of the group. However, the role of the tutor needs to be further explored in order to achieve group enthusiasm, facilitating cooperation and collaboration in the learning process. This study presents a tool that can help students achieve learning outcomes in clinical training; it has great potential in the face of increasingly virtual settings that have brought about a change in the universal teaching model. Therefore, this study addresses a gap currently existing in the literature, and brings added value to the disconnect between the university professor and the undergraduate nursing student during clinical practice in health centers. Even so, the present study has some limitations, such as validating a questionnaire that incorporates a joint evaluation construct, replicating the multicenter study in other academic courses of the nursing degree in clinical practice subjects, as well as reasoning by means of a qualitative study the differences in the mean scores obtained between the different subjects observed.

## 5. Conclusions

Adapting to the European Higher Education Area (EHEA) implies a transformation in the way university education is conceived of and implemented; new ways of assessing competencies in clinical practice courses are needed to encourage student participation.

The need to be critical and reflective is considered both a challenge and a solution to the learning and teaching processes. The hybrid model of support is a good starting point from which to encourage nursing students to develop critical thinking; it inspires them to reflect, analyze and critique, thus creating a student-centered learning environment that favors collaborative learning where they can share knowledge with their peers.

The proposed model is based on a constructivist process: students start with the detection of a problem or situation; they reflect on it among their peers in a forum mediated by a tutor; they subsequently share concept maps; and, finally, they evaluate and search for solutions in their Individual Improvement Plans.

This model is envisaged as an environment where students are actively involved in the learning process and not just passive participants. It is a space where they have the opportunity to analyze and reflect on professional practice; to understand the experience of clinical work; to be willing to change or relearn and accept responsibility for decisions made; and, above all, to aim for self-improvement. The model avoids a routine and mechanized experience of practical placements and responds to the new approach of increasingly virtualized universal education in the context of clinical practice.

## Figures and Tables

**Table 1:** IMP design.

Characteristics	Learner-focused, on their activities and reflections. Presented in electronic format through the Moodle platform. Structured and aggregated.
Objective	To encourage reflection and critical thinking among students. Formative and evaluative assessment.
Content	Six sections: reflections, proposals for improvement, how they will carry it out, with what they will evaluate their interventions, evaluation of the forum and practicum.
Assessment	Score within the clinical practice subject: 15% of the final grade.

## Data Availability

The data presented in this study are available on request from the corresponding author.

## Public Involvement Statement

Final year nursing degree students participated in this study.

## Guidelines and Standards Statement

This manu-script was drafted against the the Strengthening the Reporting of Observational Studies in Epidemiology (STROBE) guidelines were followed in this observational study.

## Appendix A

**Table A1:** Analysis of the self-administered questionnaires of final-year nursing students on the hybrid model of learning in clinical practice.

**Sociodemographic Variables**	**Academic Years:** **2018–2019 and 2019–2020** **(n = 158)**				
Sex, n (%) Man Woman	46.5 (29.4) 111.5 (70.6)				
Age, mean (SD)	21.18 (2.19)				
Questions (n = 50)	Total Nursing Students (n = 158)	Geriatric Care (n = 55)	Critical Care (n = 52)	Psychiatry and Mental Health (n = 49)	*p*-value
**In relation to the virtual learning environment and the debate forum**					
1. The didactic material has clearly explained the operation of the virtual learning space where the tutoring discussion forum is located.	3.10 (0.58)	3.20 (0.40)	3.09 (0.71)	3.00 (0.58)	0.210
2. There are materials and/or guides that contain information about the formation of objectives, content, activities to develop and evaluation.	3.22 (0.70)	3.16 (0.63)	3.33 (0.64)	3.16 (0.83)	0.355
3. The presentation has indicated the technical prerequisites.	3.21 (0.59)	3.04 (0.58)	3.41 (0.57)	3.18 (0.57)	0.004 *
4. The goals have been explicit and realistic.	3.37 (0.56)	3.23 (0.61)	3.38 (0.77)	3.32 (0.63)	0.001 **
5. There is a timetable.	3.33 (0.85)	3.42 (0.83)	3.41 (0.84)	3.14 (0.87)	0.180
6. The supervised debate forum allows sharing ideas and knowledge.	3.18 (0.78)	3.29 (0.71)	3.30 (0.74)	2.94 (0.85)	0.290
7. The virtual space of learning Moodle (has been an easy to use environment).	3.30 (0.74)	3.55 (0.60)	3.15 (0.83)	3.18 (0.70)	0.007 *
8. I have felt comfortable using the virtual learning space (Moodle).	3.45 (0.59)	3.49 (0.60)	3.48 (0.67)	3.37 (0.49)	0.507
9. The forum has allowed you to have flexibility in your organization.	3.34 (0.62)	3.42 (0.60)	3.44 (0.60)	3.12 (0.63)	0.015
10. The subjects of the forum have been topical and of academic interest.	3.23 (0.66)	3.42 (0.50)	3.24 (0.67)	3.02 (0.75)	0.008 *
11. Virtual resources (URL’s, etc.) have been relevant for the learning process.	3.36 (0.65)	3.71 (0.46)	3.24 (0.64)	3.10 (0.68)	0.000 **
12. The contents have justified to the objectives set.	2.92 (0.80)	2.76 (0.77)	3.07 (0.91)	2.92 (0.67)	0.127
13. The topics dealt with in the debate forum presented a common thread and they have related to each other.	3.08 (0.63)	3.18 (0.64)	3.09 (0.68)	2.96 (0.54)	0.195
14. I have felt comfortable using the discussion forum.	3.29 (0.64)	3.33 (0.58)	3.24 (0.75)	3.31 (0.58)	0.768
15. Access to external links has been viable from any device.	3.36 (0.72)	3.62 (0.49)	3.35 (0.80)	3.08 (0.73)	0.001 **
16. The information provided in the virtual space has been useful to me for my correct development	3.14 (0.63)	3.27 (0.56)	3.06 (0.66)	3.08 (0.67)	0.150
**In relation to the Forum’s motivational tutor**					
17. The tutor indicates how to contact him/her.	3.16 (0.64)	3.22 (0.69)	3.15 (0.68)	3.10 (0.55)	0.652
18. The tutor made me feel good.	3.50 (0.64)	3.67 (0.47)	3.43 (0.74)	3.39 (0.64)	0.041 *
19. I have established a trust relationship with my tutor.	3.66 (0.54)	3.56 (0.60)	3.74 (0.52)	3.67 (0.47)	0.225
20. The frequency of interaction with the tutor has been as frequent as I have needed.	3.53 (0.61)	3.42 (0.66)	3.63 (0.56)	3.55 (0.61)	0.193
21. The tutor took account of consolidation strategies and knowledge transfer.	3.49 (0.59)	3.44 (0.63)	3.48 (0.61)	3.55 (0.54)	0.618
22. The tutor has proposed activities to develop collaborative learning.	3.58 (0.57)	3.47 (0.50)	3.61 (0.66)	3.67 (0.52)	0.178
23. The tutor has facilitated terminology or query sources.	3.56 (0.56)	3.33 (0.61)	3.72 (0.49)	3.65 (0.48)	0.000 **
24. The tutor has proposed the co-evaluation among the students.	3.36 (0.70)	3.20 (0.70)	3.43 (0.74)	3.47 (0.62)	0.101
25. The tutor has detailed the criteria of each activity.	3.15 (0.89)	2.93 (0.98)	3.35 (0.68)	3.16 (0.94)	0.043 *
26. The tutor has clearly described the methodology and the time of delivery of the evaluation activities.	3.38 (0.67)	3.44 (0.71)	3.37 (0.73)	3.33 (0.55)	0.706
27. Tutor sent clear and short messages.	3.37 (0.65)	3.36 (0.68)	3.46 (0.66)	3.29 (0.61)	0.387
28. The tutor has used a language adapted to the forum and is understandable.	3.56 (0.55)	3.60 (0.53)	3.72 (0.49)	3.33 (0.55)	0.001 **
29. The tutor has redirected dialogues in the forum, reformulating or deepening the interventions.	3.61 (0.56)	3.47 (0.60)	3.78 (0.54)	3.33 (0.50)	0.016 *
30. At the end of the forum, the tutor has synthesized.	3.49 (0.65)	3.47 (0.60)	3.78 (0.54)	3.59 (0.58)	0.874
31. The tutor has made individual feedback.	3.46 (0.62)	3.56 (0.60)	3.43 (0.69)	3.39 (0.57)	0.314
32. In case of doubts posed in the forum, the tutor has answered.	3.11 (0.82)	3.13 (0.98)	3.04 (0.80)	3.18 (0.63)	0.660
33. The tutor has motivated the discussion.	3.16 (0.79)	3.31 (0.84)	3.02 (0.79)	3.14 (0.74)	0.160
34. The discussions proposed by the lecturer to the Forum have been aimed at achieving goals.	3.42 (0.65)	3.40 (0.66)	3.24 (0.75)	3.27 (0.60)	0.419
35. With the discussion forum motivated by the tutor, the quality of the practices has improved.	3.42 (0.65)	3.35 (0.67)	3.50 (0.67)	3.41 (0.61)	0.463
36. The tutor-led discussion forum improves the quality of clinical practice.	3.22 (0.73)	3.40 (0.60)	3.19 (0.80)	3.06 (0.75)	0.054
**In relation to the satisfaction**					
37. I am satisfied with the quality of the support received from the tutor.	3.34 (0.67)	3.25 (0.75)	3.39 (0.71)	3.37 (0.53)	0.540
38. I am satisfied with my participation in the tutoring program.	3.59 (0.55)	3.49 (0.50)	3.69 (0.54)	3.61 (0.61)	0.181
39. The work done in the forum has helped me to confront the demands of the professional world.	3.48 (0.67)	3.49 (0.60)	3.52 (0.64)	3.43 (0.76)	0.785
40. The work done in the forum has greatly influenced my motivation.	3.11 (0.73)	3.09 (0.70)	3.15 (0.79)	3.10 (0.71)	0.912
41. The work done has influenced me to increase my degree of personal safety.	3.08 (0.74)	3.11 (0.63)	3.11 (0.86)	3.00 (0.71)	0.688
42. The work done in the forum has helped increase my competence level.	2.99 (0.75)	3.04 (0.74)	3.00 (0.78)	2.94 (0.75)	0.804
43. The work done in the forum has helped to reduce the level of stress produced in the clinical practices.	2.93 (0.78)	2.87 (0.75)	3.02 (0.79)	2.90 (0.82)	0.590
44. Indicate your assessment of the discussion forum dynamized as support of your clinical practices.	2.61 (0.90)	2.47 (0.94)	2.78 (0.90)	2.57 (0.87)	0.205
45. In general, what degree of satisfaction have you had regarding learning?	2.92 (0.84)	3.35 (0.62)	2.78 (0.82)	2.59 (0.91)	0.000 **
**Self-evaluation**					
46. Do you think you have achieved the objectives of the course?	3.21 (0.65)	3.27 (0.45)	3.22 (0.74)	3.12 (0.73)	0.493
47. Do you think you have achieved your expectations?	3.58 (0.51)	3.56 (0.50)	3.69 (0.47)	3.47 (0.54)	0.096
48. The duration of the clinical practices has allowed you to achieve the objectives.	3.47 (0.56)	3.47 (0.50)	3.61 (0.56)	3.33 (0.69)	0.051
49.Did the duration of the internship allow you to achieve your objectives?	3.31 (0.71)	3.51 (0.63)	3.26 (0.78)	3.31 (0.68)	0.148
50. Did the individual improvement plan allow you to apply what you learned in the clinical practice?	3.32 (0.97)	3.47 (1.00)	3.22 (1.08)	3.27 (0.81)	0.360

Variables are presented as Mean ± standard deviation (SD) and ANOVA test is used to assess differences between groups. * *p* < 0.05; ** *p* < 0.001.
